# Evaluating Declines in Compliance With Ecological Momentary Assessment in Longitudinal Health Behavior Research: Analyses From a Clinical Trial

**DOI:** 10.2196/43826

**Published:** 2023-06-22

**Authors:** Sarah Tonkin, Julie Gass, Jennifer Wray, Eugene Maguin, Martin Mahoney, Craig Colder, Stephen Tiffany, Larry W Hawk Jr

**Affiliations:** 1 Stephenson Cancer Center Tobacco Settlement Endowment Trust Health Promotion Research Center University of Oklahoma Health Sciences Center Oklahoma City, OK United States; 2 Department of Veterans Affairs Center for Integrated Healthcare VA Western NY Healthcare System at Buffalo Buffalo, NY United States; 3 Department of Psychology The State University of New York: University at Buffalo Buffalo, NY United States; 4 Department of Psychiatry and Behavioral Sciences Medical University of South Carolina Charleston, SC United States; 5 Ralph H Johnson VA Healthcare System Charleston, SC United States; 6 Departments of Internal Medicine and Health Behavior Roswell Park Cancer Comprehensive Cancer Center Buffalo, NY United States

**Keywords:** ecological momentary assessment, compliance, health behavior, methodology, longitudinal, health behavior, smoking, smoker, cessation, quit, adherence, dropout, RCT, cigar, retention

## Abstract

**Background:**

Ecological momentary assessment (EMA) is increasingly used to evaluate behavioral health processes over extended time periods. The validity of EMA for providing representative, real-world data with high temporal precision is threatened to the extent that EMA compliance drops over time.

**Objective:**

This research builds on prior short-term studies by evaluating the time course of EMA compliance over 9 weeks and examines predictors of weekly compliance rates among cigarette-using adults.

**Methods:**

A total of 257 daily cigarette-using adults participating in a randomized controlled trial for smoking cessation completed daily smartphone EMA assessments, including 1 scheduled morning assessment and 4 random assessments per day. Weekly EMA compliance was calculated and multilevel modeling assessed the rate of change in compliance over the 9-week assessment period. Participant and study characteristics were examined as predictors of overall compliance and changes in compliance rates over time.

**Results:**

Compliance was higher for scheduled morning assessments (86%) than for random assessments (58%) at the beginning of the EMA period (*P*<.001). EMA compliance declined linearly across weeks, and the rate of decline was greater for morning assessments (2% per week) than for random assessments (1% per week; *P*<.001). Declines in compliance were stronger for younger participants (*P*<.001), participants who were employed full-time (*P*=.03), and participants who subsequently dropped out of the study (*P*<.001). Overall compliance was higher among White participants compared to Black or African American participants (*P*=.001).

**Conclusions:**

This study suggests that EMA compliance declines linearly but modestly across lengthy EMA protocols. In general, these data support the validity of EMA for tracking health behavior and hypothesized treatment mechanisms over the course of several months. Future work should target improving compliance among subgroups of participants and investigate the extent to which rapid declines in EMA compliance might prove useful for triggering interventions to prevent study dropout.

**Trial Registration:**

ClinicalTrials.gov NCT03262662; https://clinicaltrials.gov/ct2/show/NCT03262662

## Introduction

Ecological momentary assessment (EMA) uses digital technologies (eg, smartphones) to gather real-world, real-time data from research participants multiple times per day, thereby overcoming the notable problems with retrospective recall and yielding assessments with excellent temporal resolution and representation of “in the moment” participant experiences [1,2]. EMA has become a popular assessment tool in the study of health behaviors including substance use [3-5], eating and weight loss [6,7], pain [8,9], and a variety of medical illnesses [10,11]. EMA is also useful for testing hypothesized mechanisms of health behavior interventions [12-14] and long-term maintenance of behavior change [15]. For example, in an EMA study by Gwaltney et al [13], self-efficacy for quitting smoking declined in the days leading up to a smoking lapse, whereas positive smoking outcome expectancies increased the day before the lapse. Such demonstrations of EMA’s ability to capture the proximal antecedents of lapse and relapse behavior have contributed to a growing interest in using EMA to trigger just-in-time, adaptive interventions [16].

In each of these areas of health behavior research, the use and validity of EMA depend on maintaining EMA compliance across weeks or even months (see Stone and Shiffman [2], *P* 240); however, few studies have responded to calls for the evaluation of changes in EMA compliance over time [17-19] despite EMA being used over longer time frames [20-22]. Extending the logic of Stone and Shiffman [2], marked declines in compliance degrade both the temporal resolution and representativeness of the data, which threatens the valid evaluation of health behavior treatment and maintenance mechanisms [15]. Several studies suggest that such concerns are warranted, as evidenced by declines in EMA compliance over the initial days and weeks of assessment [10,14,23] (note that comparisons of overall compliance across studies of varying durations are generally insensitive to these within-study effects of time [18,24]). However, the magnitude of these declines has been quite variable, ranging from just 2% from week 1 to week 2 in adults with chronic pain [18] to declines of 10%-30% across 3-4 weeks of EMA in studies of various health behaviors among youth and young adults [10,14].

Very little is known about the time course of EMA compliance beyond a few weeks. If compliance declines at a linear rate, the health behavior studies reviewed in the prior paragraph would suggest EMA compliance drops 2%-9% per week. Whereas a 2% per week decline would likely have a modest impact on EMA data [18], a 9% per week decline would substantially reduce temporal precision for protocols lasting more than a few weeks. Even more concerning, there is preliminary evidence that the rate of EMA compliance decline may accelerate after the initial weeks of data collection [14,25], including pilot studies reporting declines as high as 90% and week-long periods of 0% compliance for the majority of the participants [20,22]. Unfortunately, these pilot studies did not use randomly scheduled prompts that are typical of EMA or report weekly compliance rates. Thus, to our knowledge, no health behavior study has systematically examined week-to-week changes in long-term EMA compliance.

This study seeks to fill this gap in the literature by examining EMA compliance rates over a 9-week period in the context of a randomized controlled clinical trial for smoking cessation among community adults. Based on the work reviewed above, we tested the competing hypotheses that EMA compliance would (1) gradually and linearly decline across weeks or (2) exhibit greater declines in later weeks compared to earlier weeks (ie, accelerating declines tested as a quadratic trend).

We also explored candidate predictors of both initial EMA compliance and changes in compliance over time to better understand variables related to assessment completion in clinical EMA samples [26]. In general, compliance may be both higher and better maintained among participants with fewer competing demands and greater flexibility in their schedules (eg, retired participants [18,24]). Although we did not directly assess competing demands, we tested whether younger age [18,27] and full-time employment predicted lower initial compliance and steeper declines across the 9-week EMA protocol. Furthermore, data collection occurred prior to and during the COVID-19 pandemic; because stay-at-home orders and remote work may have allowed for better maintenance of compliance during the pandemic, we also evaluated the COVID-19 context as a predictor of compliance.

We also examined how aspects of the EMA protocol that may have produced a greater burden and less flexibility affected compliance. Specifically, we predicted that initial EMA compliance would be higher, and declines over time would be smaller for (1) participants completing EMA on their own phone compared to a study-provided phone (as the latter often ended up carrying 2 phones, which might have increased burden for completing assessments) [24] and (2) morning assessments tailored to each participant’s schedule compared to randomly presented assessments [28]. Both phone and assessment types have been identified as possible predictors of compliance and are key considerations in the design of EMA protocols [19]. However, we are not aware of any health behavior study that has directly compared the impact of these 2 variables on EMA compliance over time. Finally, we explored EMA compliance trends over time as a function of additional study and demographic characteristics identified as important predictors of EMA compliance in prior research (treatment group, sex, race, income, and education [18,22,27-29]), and we tested if participants who eventually dropped out of the study differed in compliance rates compared to those who completed the study.

## Methods

### Participants

A total of 257 cigarette-using adults were participated in a randomized, double-blind placebo-controlled clinical trial examining extended prequit varenicline (Chantix) for smoking cessation (ClinicalTrials.gov NCT03262662 [30]). Eligibility criteria included being 18-70 years old, smoking ≥5 cigarettes per day for the past ≥6 months, and having at least moderate motivation to quit smoking. Exclusion criteria included current use of other nicotine or smoking cessation products, being pregnant or breastfeeding, having lifetime psychosis or bipolar disorder or currently using antipsychotics, currently having major depression, and having any contraindicated medical condition or medication determined by the study physician (see ClinicalTrials.gov for further details). See Table 1 for participant characteristics. Due to a data cleaning error, EMA compliance was missing for 7 participants across 3 weeks (<1% of compliance data). Therefore, correcting this error and including these missing data did not substantively change the present results.

**Table 1:** Participant demographic information^a^.

Characteristics		Values
Sex (male:female), n:n		112:145
Age (years), mean (SD)		53.67 (10.1)
**Race, n (%)**		
	White	191 (75.6)
	Black or African American	54 (21.1)
	Other race	11 (4.3)
**Employment status, n (%)**		
	Working full-time	114 (53.7)
	Working part-time, unemployed, retired, disabled, or homemaker	132 (46.3)
**Household income (US $), n (%)**		
	<50,000	106 (41.3)
	>50,000	127 (49.4)
**Education, n (%)**		
	<12 years (high school degree/General Educational Development or less)	60 (24.3)
	>12 years (some college degree and beyond)	187 (75.7)

^a^Race (n=1), employment (n=11), income (n=9), and education (n=10) information were not collected from some participants. Employment, income, and education questions were not included in the initial demographic form used in this study. An additional 15 (6%) participants chose not to disclose to their income, resulting in 233 for the income variable.

### Procedures

The sample included participants who met trial eligibility criteria and attended the first treatment visit where half of the sample was randomized to a standard course of varenicline treatment (12 weeks initiated 1 week prior to their target quit day) and half to an extended course (15 weeks initiated 4 weeks prior to their target quit day). Participants in the standard course received a placebo during the 3 weeks prior to varenicline initiation. Participants received up to 6 in-person smoking cessation counseling sessions during the 9-week EMA protocol (see [30] for details).

Daily data collection was completed using mobile EMA software (ilumivu), a smartphone-based EMA platform that operates on Android and Apple devices. Participants could use their personal smartphone or a loaned Samsung Galaxy “study phone” running on Android. Assessment schedules were based on the participant’s usual wake-up time. Participants completed a brief (~15 minutes) training including an overview of the application and a practice assessment. Participants were given a “frequently asked questions” handout and a “check-in” call was completed during the first week of the EMA period (the baseline week) for troubleshooting and compliance review. Technical problems and compliance were also reviewed at each in-person visit (up to 7 visits occurring every 1-2 weeks), and participants could call the study office if they had any technical problems.

Two types of brief EMA were administered: morning assessments (1 per day) and random assessments (4 per day). Participants earned US $1 per assessment completed and were paid at each in-person visit. To be eligible for the study, participants were required to achieve 40% compliance for morning assessments and 50% for random assessments during the baseline week. Participants who did not meet the minimum compliance completed a second baseline week (n=17) and were excluded if they continued to fall below the requirement (n=8; only the second baseline week data were included in the present analyses for participants who were eligible after the second attempt). During the study, the eligibility criterion for the random assessments was dropped to reduce participant burden; 27% (n=70) of participants in this sample were recruited after this criterion was removed. A supplemental analysis suggested that removing this eligibility criterion did not contribute to significant differences in compliance rates (*P*>.31). After the baseline week, eligible participants completed 8 weeks of EMA with no minimum compliance requirements (9 weeks total). See Multimedia Appendix 1 for further details on the procedures of the parent trial.

### Measures

#### Morning Assessments

Participants were instructed to complete the morning assessment within 60 minutes of waking and before their first cigarette of the day (if still smoking). The morning assessments were available 1 hour before and 2 hours following the participant’s usual wake time. See Multimedia Appendix 1 for details on measures administered during assessments (data to be presented in a subsequent paper). On average, participants completed the morning assessment in 1.7 (SD 1.1) minutes.

#### Random Assessments

Participants received four pseudorandomly timed survey prompts. The random assessments occurred over a 12-hour window divided into four 3-hour blocks with 1 assessment occurring in each block. The first block began 2 hours after the participant’s usual wake time. Participants received an initial notification and 2 reminder notifications to complete each random assessment. Surveys had to be initiated within 15 minutes of the initial notification and completed within 5 minutes of initiation. On average, the random assessments took 1.1 (SD 0.7) minutes to complete.

### Data Reduction

Of the 320 participants who met the baseline compliance eligibility criteria and attended the first treatment visit, 60 participated in an optional substudy that varied the frequency and amount of reinforcement for completing EMA (data to be presented in a separate report), and 3 participants were removed from the sample due to software problems with the mobile EMA app (data loss from a server crash), leaving a sample size of 257. Race, employment status, household income, and education were dichotomized as in the primary outcome paper of the parent trial [30].

Within-subject percent compliance was computed for each of the 9 weeks, separately for morning assessments (up to 7 observations per week on each participant) and random assessments (up to 28 observations per week on each participant). Thirty (12%) participants dropped out of the study (withdrew or withdrawn or missed 3 visits in a row) during the EMA period. These participants were retained in the final sample, with data after their final week of study participation set to missing (not 0), and study dropout status (dropped out or completed) was examined as a predictor of EMA compliance rates.

### Data Analysis

All analyses were conducted in SPSS (version 28; IBM Corp). A series of multilevel models (MLMs) were estimated to assess changes in weekly EMA compliance over time and to evaluate participant and study characteristics as predictors of compliance. Morning and random assessment compliance rates were analyzed in the same model to allow direct comparisons between assessment types. This yielded a data set with up to 18 repeated measures per participant (2 assessment types×9 weeks).

First, a series of unconditional MLMs were used to establish the basic shape of trajectories of change. We started with an intercept-only model (with random intercept) to estimate the interclass correlation coefficient (ICC), then linear and quadratic slopes were added to examine the shape of change. Unconditional models were run using a maximum-likelihood estimator to compare model fit using likelihood ratio tests and to determine the structure of random effects. All other models used the default estimator (restricted maximum likelihood).

Second, after determining the best fitting model to characterize change, predictors of compliance were evaluated. Assessment type was included in all predictor models. Additional predictors were mean-centered age, employment status (full-time vs not full-time, which included part-time employed, unemployed, retired, homemakers, and disabled participants), EMA phone type (study phone vs participant’s personal phone), COVID-19 context (enrolled before vs after March 22, 2020), treatment group (extended vs standard varenicline run-in), as well as additional participant demographics, including race (Black or African American vs White), sex (male vs female), income (<US $50,000 vs ≥ US $50,000 yearly), education (high school or less vs some college or greater), and study dropout (study completers vs study dropouts). Predictors were first considered in separate individual models evaluating the first-order effect of the predictor, the interaction with the slope over time, the interaction with assessment type (morning vs random assessments), and the 3-way interaction between the predictor, slope, and assessment type. After assessing predictors of compliance individually, a single model including all significant effects from the individual models assessed the unique effects of each predictor on EMA compliance. Simple slopes and effects were used to probe significant interactions [31]. Since the continuous compliance outcome is bounded by 0 and 1, models were rerun as a series of beta regressions with random effects using Proc Glimmix (SAS) [31]. Results were generally robust across these modeling choices and only the MLM models are presented (see Multimedia Appendix 1).

### Ethics Approval

Procedures were approved by the University at Buffalo’s institutional review board (IRB ID:RNI00000386).

## Results

### Baseline Model: Unconditional MLMs

Table S2 in Multimedia Appendix 1 presents a summary of the results for the unconditional models, including *P*-values and parameter estimates. An initial, random intercept-only model suggested that compliance across all participants, weeks, and collapsing across assessment types was 66%. The ICC estimate for the random intercept supported individual differences in compliance rates (ρ=0.34; *P*<.001). That is, 34% of the variance in compliance rates was attributable to individual differences, which indicated a multilevel approach was appropriate.

**Figure 1:**
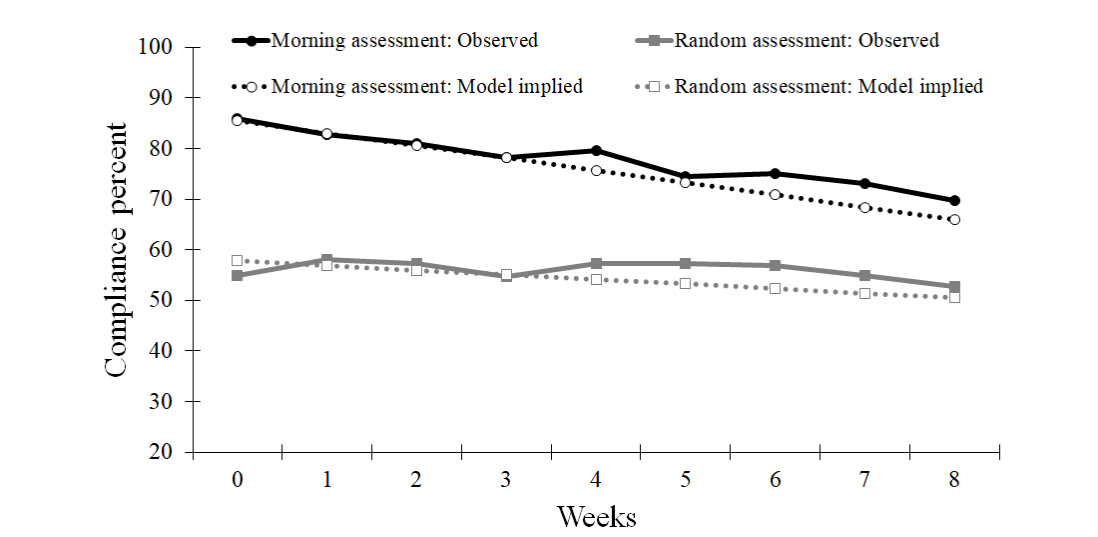
Observed and model implied morning and random assessment compliance trends across the 9-week ecological momentary assessment period.

Adding a random linear slope (with intercept representing the baseline week of the EMA period) improved model fit (*χ*^2^_2_=185.72; *P*<.001). The fixed effect of the linear slope suggested that, on average, compliance rates steadily declined (see Table S2 in Multimedia Appendix 1 and Figure 1). Moreover, the random linear slope was statistically significant (*P*<.001), suggesting individual differences in rates of change. Adding a random quadratic trend improved model fit over the random linear slope model (*χ*^2^_4_=24.44; *P*<.001). However, the quadratic slope mean (β=−.001; *P*=.25) and variance estimates (σ^2^=0.00002; *P*=.06) were not significantly different from 0. Based on these results, subsequent models included the random intercept and random linear slope but not the quadratic slope to improve parsimony.

### Compliance Predictor Models: Conditional MLMs

The results for the individual models can be found in Table S4 in Multimedia Appendix 1. The assessment type showed a significant first-order effect and interacted with the linear slope (see Table 2). Compliance during the EMA baseline period (week 0) was higher for morning assessments (81%) compared to random assessments (52%). As seen in Figure 1, compliance dropped for both morning and random assessments, but this decline was greater for morning assessments (−2% per week) than for random assessments (−1% per week). The random effect of assessment type was significant (*P*<.001), suggesting significant individual variability in the effect of assessment type. Therefore, the random effect of assessment type was included in all subsequent models to examine which participant and study variables contributed to differences in the completion rate of morning assessments compared to random assessments. All subsequent models examined the first-order effect of the predictor, its interaction with the linear slope, its interaction with the assessment type, and the 3-way interaction between the predictor, slope, and assessment type. Next, employment status, study dropout, mean-centered age, phone used, race, sex, income, education, COVID-19 context, and treatment group were tested in individual models with the random effect of assessment type and linear slope. Only employment, study dropout, and age were related to changes in EMA compliance over time (see Table 2).

**Figure 2:**
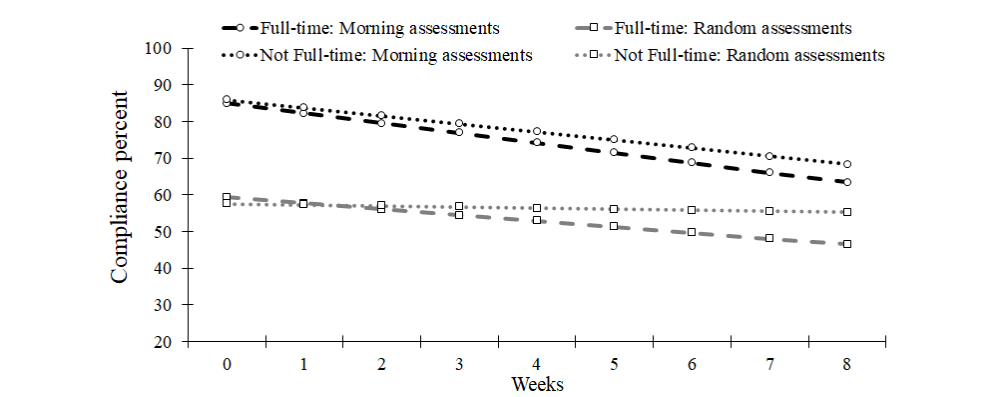
Model implied morning and random assessment compliance trends across the 9-week ecological momentary assessment period for participants employed full-time and those not employed full-time (eg, part-time employment, retired, and disabled).

A 3-way interaction between employment, assessment type, and slope was significant (see Figure 2). Declines in morning assessment compliance were observed for both participants working full-time (−3% per week) and participants not working full-time (−2% per week). However, random assessments declines were only significant for participants working full-time (−2% per week), while compliance did not change across weeks for those not working full-time (*P*=.43). The first-order effect of employment (*P*=.79) and its 2-way interactions with assessment type (*P*=.41) and slope were nonsignificant (*P*=.68).

**Figure 3:**
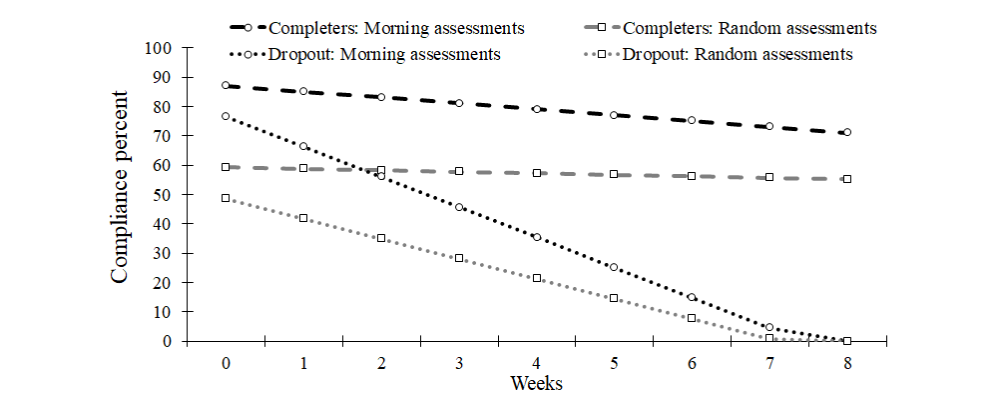
Model implied morning and random assessment compliance trends across the 9-week ecological momentary assessment (EMA) period for participants that completed the EMA period and participants that dropped out.

A 3-way interaction between study dropout, assessment type, and slope was significant (see Table 2 and Figure 3). Compliance declined more quickly across weeks among participants who eventually dropped out with morning assessments declining at a rate of 10% per week and random assessments declining at a rate of 7% per week. In comparison, among study completers, morning assessments declined at a rate of 2% per week and random assessments remained relatively consistent across weeks (−0.5%; *P*=.06). The first-order effect of study dropout was also statistically significant; study completers had higher initial compliance rates during the baseline week (morning assessments=87%; random assessments=60%), compared to participants who later dropped out (morning assessments=77%; random assessments=49%).

**Figure 4:**
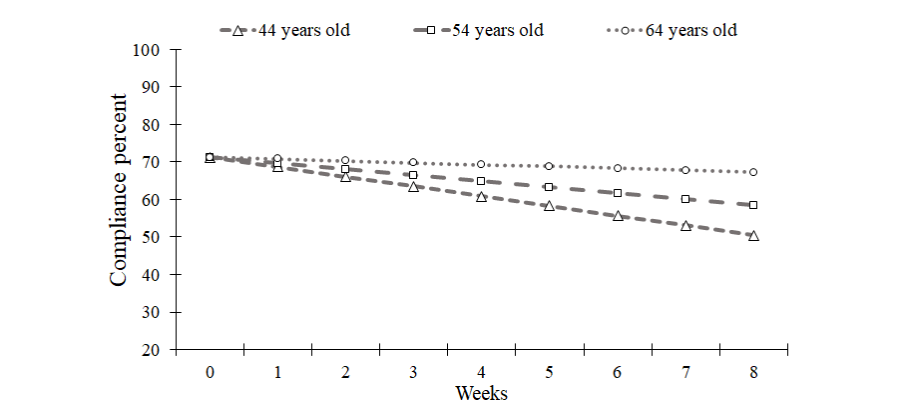
Model implied trends for overall compliance across the 9-week ecological momentary assessment period at the mean age of the sample (54 years old) and at 1 SD below (44 years old) and above (64 years old) the mean.

A 2-way interaction between age and slope was significant. As seen in Figure 4, simple slope analyses suggested compliance rates declined less steeply for older participants (64 years old; −1.5% per week) compared to younger participants (44 years old; −3.4% per week). Age also interacted with assessment type. During the baseline week, younger participants had lower morning assessment compliance compared to older participants (younger than 45 years=82%; 45-64 years old=86%; older than 64 years=89%), whereas the opposite was true for random assessment compliance (younger than 45 years=61%; 45-64 years old=60%; older than 64 years=55%).

**Table 2:** Results summary and parameter estimates for the multiple predictor model.

Multiple predictor model	β (SE)	*P* value
**Intercept**	.81 (0.03)^a^	<.001^a^
**Slope**	−.02 (0.00)^a^	<.001^a^
**Assessment type**	−.29 (0.02)^a^	<.001^a^
**Assessment×slope**	.02 (0.00)^a^	<.001^a^
Age	.00 (0.00)	.47
Employment	−.01 (0.03)	.79
**Race**	.08 (0.02)^a^	.001^a^
**Study drop out**	−.11 (0.04)^a^	.01^a^
COVID context	.00 (0.02)	.92
**Age×assessment**	−.003 (0.00)^a^	<.001^a^
Employment×assessment	.02 (0.03)	.41
Study dropout×assessment	−.02 (0.04)	.68
*COVID-19 context×assessment*	*.03 (0.02)* ^b^	*.18* ^b^
**Age×slope**	.001 (0.00)^a^	<.001^a^
Employment×slope	.00 (0.01)	.68
**Study dropout×slope**	−.08 (0.01)^a^	<.001^a^
**Employment×assessment×slope**	−.01 (0.00)^a^	.03^a^
**Study dropout×assessment×slope**	.02 (0.01)^a^	.04^a^

^a^Effects that remained significant in both the individual and multiple predictor models.

^b^Italicized items denote effects that were significant in the individual models but nonsignificant in the multiple predictor model.

The first-order effect of race was statistically significant; overall compliance among White participants and Black or African American participants was 67% and 61%, respectively. Sex, phone type, education, income, and treatment group did not produce any statistically significant effects on EMA compliance. The COVID-19 context interacted with assessment type; random assessment compliance was modestly higher during the COVID-19 pandemic (+3%), while morning assessment compliance was modestly lower (−2%). However, this effect was nonsignificant after accounting for the other predictors, suggesting the observed relationship between the COVID-19 context with EMA compliance may be better explained by other variables.

## Discussion

### Principal Findings

Building on prior short-term EMA studies of health behavior [18,24] using an extended EMA assessment period in a large sample, we systematically examined week-to-week changes in compliance over a 9-week period in the context of a smoking cessation trial. We examined baseline levels of compliance at the start of the assessment period, the overall rate of change in compliance over time, and a range of potential predictors of EMA compliance within a clinical sample. Baseline compliance was higher for scheduled morning assessments, with stronger linear rates of declines compared to random assessments. Declines in compliance were stronger for participants who were younger, employed full-time, and dropped out of the study. Overall compliance was higher among White participants.

A methodological concern for EMA studies lasting several months is markedly lower EMA compliance occurring in the latter weeks of assessment, which could seriously undermine the validity of those data [2]. Fortunately, this concern was not realized. Although the decline in EMA compliance across weeks was statistically significant, the trend was linear and modest in magnitude (1%-2% per week; Figure 1). Additionally, among morning assessments which showed stronger declines (2% per week), baseline compliance was higher (81%), lessening concerns regarding some data loss over time. While this bodes well for the validity of long-term EMA studies of health behavior [15], specific aspects of the study design and sample (eg, frequent participant contact and highly motivated participants receiving treatment) may have increased compliance and limited the generalizability of these findings. However, the results of a recent meta-analysis suggest most study characteristics are unrelated to EMA completion rates, except that monetary incentives improve compliance [32]. Although strong conclusions require replication, the data from the present smoking cessation trial suggest that, on average, compliance with paid EMA protocols typical of health behavior studies (67%-85%; see [14,18,19,23,24,27]) can be adequately maintained for periods of at least 2 months. It is important both to discuss the degree to which the overall pattern of EMA compliance was predicted by several variables and to more explicitly consider operationalizations of “adequate” compliance.

### Predictors of EMA Compliance

As hypothesized, initial compliance was substantially higher for the morning assessments than for the random assessments (see Figure 1). Even though compliance with morning assessments declined across weeks at twice the rate of random assessments (2% per week and 1% per week, respectively), average compliance during the final week of EMA remained 17% higher for morning assessments compared to random assessments. Higher compliance rates for morning assessments are likely due to these prompts being tailored and fixed to a 3-hour window around the participant’s typical wake time, allowing for greater flexibility in completing these assessments. However, since compliance was near the ceiling, morning assessments had greater room to fall (eg, the law of initial values [33]). Additionally, morning assessments were fixed and predictable, which may have enhanced habituation occurring in response to repeated EMA prompts [34]. In contrast, the random assessments were momentary in nature. By definition, these prompts were less predictable and designed to capture reports during much briefer windows (ie, initiated within 15 minutes of the initial alarm and completed within 5 minutes), which may have attenuated declines related to habituation but also reduced overall compliance rates [28].

Based on the present results and prior literature, compliance can be improved for random assessments by expanding the assessment window, using less stringent time-to-initiation/completion criteria, or incorporating a “snooze” feature for delaying initiating the survey [35]. However, such features allow for greater participant control over the timing of the “moments” that are sampled. Our approach emphasized sampling of random, brief windows, to minimize the opportunity for the participant to change the nature of the moment being assessed (eg, waiting until they are less stressed to complete the “random” assessment). While the trade-off between increasing participant compliance and maintaining random momentary assessments is difficult to balance, parametric work may provide valuable insights about the impact of such EMA time-to-initiation or -completion criteria both on compliance and data quality [36].

As predicted, the rate of decline in EMA compliance was negatively associated with participant age. Despite younger participants having higher rates of random assessment compliance at the start of the EMA period, compliance in the final week was approximately 20% higher for older participants (64 years old) than for younger participants (44 years old; see Figure 4). Furthermore, older age was associated with higher baseline compliance with the scheduled morning assessments, which may have contributed to higher compliance rates. The present data from cigarette-using adults enrolled in a treatment study are consistent with the results of a meta-analysis of shorter-term studies in the pain literature [18]. More generally, the marked between-study variability in the degree to which EMA compliance declines across time may reflect age differences across studies. That is, health behavior studies reporting EMA compliance declines of 10%-30% across 3-4 weeks of EMA have tended to focus on youth and young adults [10,14,25].

We initially conceptualized age as a predictor based on the perspective that compliance would be both higher initially and better maintained among participants with fewer competing demands and greater flexibility in their schedules (eg, retired participants [18,24]). Indeed, we observed some support for this explanation. Most notably, those not working full-time maintained better compliance rates across weeks. This was particularly true among random assessments. The timing of EMA around specific windows likely impacts compliance for individuals employed full-time since prompts occurring during flexible hours (eg, lunchtime and outside of meetings) are the most likely to be answered [37]. Future work may consider how compliance is related to the timing of regularly scheduled events (eg, meals and commutes). However, other variables assessed in this study that we believed to be associated with greater flexibility, including the use of a personal smartphone and the COVID-19 context, were not associated with EMA compliance. More detailed assessment of daily conflicts or flexibility may help clarify variability in EMA compliance and elucidate methods to improve compliance among participants with particularly demanding, inflexible schedules.

The most striking declines in EMA compliance (7%-10% per week) occurred among participants who eventually dropped out of the study. It is important to note that EMA compliance was coded as missing (not 0%) once a participant was no longer in the study. Consequently, declines in EMA compliance preceded study dropout. It seems plausible that either the burden of EMA contributed directly to study dropout or third variables (eg, general motivation or stress) contributed to both poor EMA adherence and study dropout. Either way, future studies may seek to replicate the relationship between study attrition and EMA compliance. Detection of early noncompliance or rapid declines in EMA compliance may be helpful for identifying participants who need extra support to be retained in long-term health behavior studies, a novel twist on using EMA to trigger just-in-time, adaptive interventions [16].

Consistent with other smoking research, compliance was lower among African American participants than among White participants [29]. However, other racial categories and Hispanic or Latinx ethnicity could not be explored as predictors of compliance due to small sample sizes. On average, compliance was 6% lower for African Americans, raising concerns that EMA methods may be less effective in capturing the experiences of these participants. Differences in daily stress and mistrust attributed to institutional racism may have contributed to the observed differences in compliance. For example, marginalized populations may have schedules that offer less flexibility for completing EMA, or following repeated mistreatment may be understandably less motivated to share their momentary experience [38,39]. This is of concern, given the need to address racial disparities in a wide range of health behaviors and outcomes. Additional methodological and community-based work is needed to develop a more complete understanding of race-related issues in EMA research including competing demands due to systemic racial barriers, as well as culturally informed assessments to more accurately capture and represent the experiences of racialized groups [40].

Importantly, the predictors of EMA compliance discussed above (except for the COVID-19 context) all remained significant when included in a single, multiple predictor model, demonstrating that the effects discussed above were each uniquely and additively predictive of EMA compliance. However, because the effects are additive, it is also reasonable to consider them in combination. For example, the present findings suggest EMA compliance would be particularly low among younger African Americans who work full-time. Given the need to better capture the experiences of marginalized groups described above, these findings raise concerns about adequate representation in EMA data across individuals. This, in turn, raises the question: what constitutes “adequate” EMA compliance?

### “Adequate” EMA Compliance

What level of EMA compliance should we strive for in long-term studies of health behavior and at what level is the validity of EMA seriously threatened? Certainly, when there are long periods of 0% compliance in the majority of the sample [22], EMA provides little temporal precision and minimal representation of real-world, real-time participant experience. Fortunately, that was not the case in the present study, despite a 9-week study period. However, the overall compliance rate in the present work, as in many other EMA studies of health behavior [17,18,24,26], fell short of the traditional recommendation of an 80% threshold for “adequate” compliance [2]. Average EMA compliance rates were 77% for morning assessments (~5 out of 7 assigned per week; SD 19%) and 55% for random assessments (~15 of 28 assigned per week; SD 20%). Importantly, it is becoming increasingly clear that a single number does not adequately describe EMA compliance. Trull and Ebner-Priemer [19] recommended that compliance be reported by assessment type (eg, morning vs random assessments) and time interval (we recommend reporting by day in shorter-duration studies and by week for longer durations).

Ultimately, “acceptable” compliance depends in large part on the particular questions being asked and analyses being performed. Greater temporal precision and representativeness of momentary changes is needed to test complex within-day mediational analyses involving changes in multiple processes (eg, affect, self-efficacy, and momentary changes in health behavior) than to examine week-to-week changes in health behavior and related processes. However, even for complex mediational analyses, statistical approaches to address missing data (eg, imputation strategies and full-information estimation) can mitigate bias even at high levels of missingness [41]. However, such corrections are enhanced when predictors of missing data are included [42].

These results may be useful in optimizing approaches to deal with missing EMA data and addressing issues of noncompliance. Our findings suggest adding variables associated with compliance including assessment type, age, employment status, and race into statistical models can help to reduce bias from missing data. Additional quantitative and qualitative work is also needed to better understand the mediating mechanisms that cause these groups to have reduced compliance (eg, increased demands, lowered research engagement, and stress). Such information can help inform EMA protocols to better meet the needs of participants and increase compliance.

### Summary

EMA has allowed health behavior researchers to collect large amounts of real-time, real-world data with excellent temporal precision, providing insights into the processes that drive health behavior change and maintenance over weeks or even months. The present study provided an initial evaluation of the degree to which compliance, critical for the validity of EMA, is maintained over 9 weeks in the context of a randomized controlled trial for smoking cessation among community adults. Primary findings generally replicated and extended prior work attempting to identify compliance predictors in clinical samples [26] and with shorter protocols in the pain literature [18]: on average, EMA compliance declined modestly and linearly over the 9-week period (see Figure 1).

Together with prior work, these data suggest that, under most circumstances and for most participants, EMA can reasonably be used to monitor health behavior and related processes over periods of at least 2 months. However, the rate of decline was greater among younger people, people employed full-time, and particularly among people who eventually dropped out of the study. The present data call attention to the need to develop targeted strategies for maintaining long-term EMA compliance, including further work on the mechanistic processes that drive compliance; conversely, the data raise the possibility that marked declines in EMA compliance over time may be useful triggers of just-in-time, adaptive interventions for enhancing retention in long-term studies of health behavior.

## Appendix

Multimedia Appendix 1 Supplemental materials.

## Abbreviations

EMA: ecological momentary assessment
ICC: interclass correlation coefficient
MLM: multilevel model
