# Supplementary material for: Evaluating Declines in Compliance With Ecological Momentary Assessment in Longitudinal Health Behavior Research: Analyses From a Clinical Trial
Source: J Med Internet Res. 2023 Jun 22;25:e43826. doi: 10.2196/43826 (PMC10337346; doi:10.2196/43826)
Supplement: Multimedia Appendix 1 [file jmir_v25i1e43826_app1.docx]

**EMA Surveys and Randomization**

Throughout the study, participants had the opportunity to complete one morning assessment and four random assessments each day. Three types of morning assessments (Morning Assessment 1, Morning Assessment 2, and Morning Assessment 3; AM 1, 2, and 3 in Table S1) and two types of random assessments (Pre-Quit Random Assessment and Post-Quit Random Assessment) were presented to participants to coincide with their current phase of the study (see Table S1). As seen in Table S3, the questions presented across the different assessment types varied to match the medication phase and the pre/post-quit period. Participants were randomly assigned to either extended run-in varenicline (4 weeks of varenicline prior to the target quit day) or a standard run-in (3 weeks of placebo and 1 week of varenicline prior to the target quit day) with the end of Week 4 marking their target quit day. To reduce participant burden, questionnaires within the assessments were randomized so participants did not answer all questions at each prompt. Number of questions per assessment typically ranged from 10 to 23.

**Table S1.** Study phases and corresponding assessment types occurring within each phase. Medication was initiated during the Manipulation Phase at the start of Week 2 with half of participants assigned extended run-in varenicline and the other half assigned placebo. All participants received varenicline at the start of Week 5 during the open label phase. Target quit day occurred at the end of Week 5. EMAs began at Week 1 and ended on the last day of Week 9.

| **Week 1** | **Week 2** | **Week 3** | **Week 4** | **Week 5** | | **Week 6** | | **Week 7** | **Week 8** | **Week 9** |
| --- | --- | --- | --- | --- | --- | --- | --- | --- | --- | --- |
| Baseline | Manipulation Phase | | | | Open Label Phase | | | | | |
| Pre-Quit | | | | | | | Post-Quit | | | |
| AM 1 | AM 2 | | | | AM 3 | | | | | |
| Pre-Quit Random Assessment | | | | | | | Post-Quit Random Assessment | | | |

**Table S2.** Results summary and parameter estimates for each unconditional multilevel model.

| Unconditional Models | β (SE) | *P*-value |
| --- | --- | --- |
| Random Intercept Only |  |  |
| Intercept | 0.66 (0.01) | < .001 |
| Random Intercept, Fixed Linear Slope |  |  |
| Intercept | 0.71 (0.01) | < .001 |
| Linear Slope | -0.01 (0.00) | < .001 |
| Random Intercept, Random Linear Slope |  |  |
| Intercept | 0.71 (0.01) | < .001 |
| Linear Slope | -0.02 (0.00) | < .001 |
| Random Intercept, Fixed Quadratic Slope |  |  |
| Intercept | 0.70 (0.01) | < .001 |
| Linear Slope | -0.01 (0.01) | .14 |
| Quadratic Slope | 0.00 (0.00) | .06 |
| Random Intercept, Random Quadratic Slope |  |  |
| Intercept | 0.71 (0.01) | < .001 |
| Linear Slope | -0.01 (0.01) | .08 |
| Quadratic Slope | 0.00 (0.00) | .25 |

|  | Morning Assessment 1 | Morning Assessment 2 | | | Morning Assessment 3 | | | | | Pre-Quit | | | Post-Quit | | |
| --- | --- | --- | --- | --- | --- | --- | --- | --- | --- | --- | --- | --- | --- | --- | --- |
|  | # cigarettes yesterday | | # cigarettes yesterday | | | # cigarettes yesterday | | | | | # cigarettes past 2 hours | | | | # cigarettes past 2 hours |
| 2 out of 3 |  | |  | | |  | | | | | If 0 cigarettes: | | | | If 0 cigarettes: |
|  | Craving Scale | | Craving Scale | | | Craving Scale | | | | | Craving Scale + Future Abstinence Self Efficacy | | | | Craving Scale + Current Abstinence Self Efficacy |
|  | Minnesota Tobacco Withdrawal Scale | | Minnesota Tobacco Withdrawal Scale | | | Minnesota Tobacco Withdrawal Scale | | | | | Minnesota Tobacco  Withdrawal Scale | | | | Minnesota Tobacco  Withdrawal Scale |
|  | GI distress subscale of the Nausea Profile | | GI distress subscale of the Nausea Profile | | | GI distress subscale of the Nausea Profile | | | | | GI distress subscale of the  Nausea Profile | | | | GI distress subscale of the  Nausea Profile |
| 1 out of 2 |  | | Medication adherence/ Perceived treatment assignment | | | Medication adherence | | | | |  | | | |  |
|  |  | | Treatment outcome expectancies survey | | | Treatment outcome expectancies survey | | | | |  | | | |  |
|  | | | |  | | |  | | | | If 1+ cigarettes: | | | If 1+ cigarettes: | |
|  | | |  | | | | |  | **2 out of 4** | | | Time of last cigarette and amount of cigarette smoked | | | Time of last cigarette and amount of cigarette smoked |
|  | | |  | | | | |  |  |  |  | Modified Cigarette Evaluation Questionnaire | | | Modified Cigarette Evaluation Questionnaire |
|  | | |  | | | | |  |  |  |  | Craving Scale + Future Abstinence Self Efficacy | | | Craving Scale + Current Abstinence Self Efficacy item |
|  | | |  | | | | |  |  |  |  | Minnesota Tobacco Withdrawal Scale | | | Minnesota Tobacco Withdrawal Scale |
|  | | |  | | | | |  |  |  |  | GI distress subscale of the Nausea Profile | | | GI distress subscale of the Nausea Profile |

**Table S3.** Questionnaires and randomization included in each assessment type: Craving Scale (4 item [1]); the Minnesota Tobacco Withdrawal Scale (8 item [2]), the gastrointestinal (GI) distress subscale of the Nausea Profile (5 item [3]), Treatment Outcome Expectancies (6 item [4]), Abstinence Self-Efficacy (1 item [5]), and Modified Cigarette Evaluation Questionnaire (12 item [6]).

**Table S4.** Results summary and parameter estimates for each individual predictor model.

| Individual Models | β (SE) | *P*-value |
| --- | --- | --- |
| Assessment Type |  |  |
| Intercept | 0.86 (0.01) | < .001 |
| Linear Slope | -0.02 (0.00) | < .001 |
| Assessment Type | -0.28 (0.01) | < .001 |
| Assessment × Slope | 0.02 (0.00) | < .001 |
| Age |  |  |
| Intercept | 0.86 (0.01) | < .001 |
| Linear Slope | -0.02 (0.00) | < .001 |
| Assessment Type | -0.28 (0.01) | < .001 |
| Assessment × Slope | 0.02 (0.00) | < .001 |
| Age | 0.00 (0.00) | .13 |
| Age × Slope | 0.001 (0.00) | .01 |
| Age × Assessment | -0.004 (0.00) | .002 |
| Age × Assessment × Slope | 0.00 (0.00) | .15 |
| Sex |  |  |
| Intercept | 0.86 (0.02) | < .001 |
| Linear Slope | -0.03 (0.00) | < .001 |
| Assessment Type | -0.26 (0.02) | < .001 |
| Assessment × Slope | 0.01 (0.00) | .01 |
| Sex | -0.02 (0.03) | .45 |
| Sex × Slope | 0.00 (0.01) | .68 |
| Sex × Assessment | -0.05 (0.03) | .06 |
| Sex × Assessment × Slope | 0.00 (0.00) | .15 |
| Income |  |  |
| Intercept | 0.85 (0.02) | < .001 |
| Linear Slope | -0.02 (0.00) | < .001 |
| Assessment Type | -0.29 (0.02) | < .001 |
| Assessment × Slope | 0.02 (0.00) | < .001 |
| Income | 0.00 (0.03) | 1.00 |
| Income × Slope | 0.00 (0.01) | .86 |
| Income × Assessment | 0.03 (0.03) | .21 |
| Income × Assessment × Slope | -0.01 (0.00) | .09 |
| Employment |  |  |
| Intercept | 0.86 (0.02) | < .001 |
| Linear Slope | -0.02 (0.00) | < .001 |
| Assessment Type | -0.28 (0.02) | < .001 |
| Assessment × Slope | 0.02 (0.00) | < .001 |
| Employment | -0.01 (0.03) | .70 |
| Employment × Slope | 0.00 (0.01) | .41 |
| Employment × Assessment | 0.03 (0.03) | .28 |
| Employment × Assessment × Slope | -0.01 (0.00) | .03 |
| Race |  |  |
| Intercept | 0.81 (0.03) | < .001 |
| Linear Slope | -0.02 (0.01) | < .001 |
| Assessment Type | -0.32 (0.03) | < .001 |
| Assessment × Slope | 0.02 (0.00) | < .001 |
| Race | 0.06 (0.03) | .04 |
| Race × Slope | -0.01 (0.01) | .31 |
| Race × Assessment | 0.05 (0.03) | .08 |
| Race × Assessment × Slope | -0.01 (0.01) | .30 |
|  |  |  |
|  |  |  |
| Individual Models | **β (SE)** | ***P*-value** |
| Study Phone |  |  |
| Intercept | 0.84 (0.02) | < .001 |
| Linear Slope | -0.03 (0.00) | < .001 |
| Assessment Type | -0.27 (0.02) | < .001 |
| Assessment × Slope | 0.02 (0.00) | < .001 |
| Study Phone | 0.04 (0.02) | .12 |
| Study Phone × Slope | 0.00 (0.01) | .45 |
| Study Phone × Assessment | -0.04 (0.03) | .16 |
| Study Phone × Assessment × Slope | 0.00 (0.00) | .57 |
| Study Drop Out |  |  |
| Intercept | 0.87 (0.01) | < .001 |
| Linear Slope | -0.02 (0.00) | < .001 |
| Assessment Type | -0.28 (0.01) | < .001 |
| Assessment × Slope | 0.02 (0.00) | < .001 |
| Study Drop Out | -0.11 (0.04) | .007 |
| Study Drop Out × Slope | -0.08 (0.01) | < .001 |
| Study Drop Out × Assessment | 0.00 (0.04) | .95 |
| Study Drop Out × Assessment × Slope | 0.02 (0.01) | .05 |
| Education |  |  |
| Intercept | 0.87 (0.03) | < .001 |
| Linear Slope | -0.02 (0.01) | < .001 |
| Assessment Type | -0.28 (0.03) | < .001 |
| Assessment × Slope | 0.02 (0.00) | < .001 |
| Education | -0.02 (0.03) | .56 |
| Education × Slope | -0.01 (0.01) | .17 |
| Education × Assessment | 0.01 (0.03) | .84 |
| Education × Assessment × Slope | 0.00 (0.01) | .36 |
| COVID Context |  |  |
| Intercept | 0.86 (0.02) | < .001 |
| Linear Slope | -0.03 (0.00) | < .001 |
| Assessment Type | -0.30 (0.02) | < .001 |
| Assessment × Slope | 0.02 (0.00) | < .001 |
| COVID Context | -0.03 (0.03) | .32 |
| COVID Context × Slope | 0.00 (0.01) | .71 |
| COVID Context × Assessment | 0.06 (0.03) | .02 |
| COVID Context × Assessment × Slope | -0.01 (0.00) | .26 |
| Treatment Group |  |  |
| Intercept | 0.92 (0.04) | < .001 |
| Linear Slope | -0.03 (0.01) | < .001 |
| Assessment Type | -0.32 (0.04) | < .001 |
| Assessment × Slope | 0.02 (0.01) | .005 |
| Treatment Group | -0.04 (0.02) | .08 |
| Treatment Group × Slope | 0.00 (0.01) | .57 |
| Treatment Group × Assessment | 0.03 (0.02) | .27 |
| Treatment Group × Assessment × Slope | 0.00 (0.00) | .74 |

**Beta Regressions**

Although the compliance outcome is continuous, it is bounded by 0 and 1, and there is some concern that using a general linear mixed model may be not be appropriate. Accordingly, we reran our models as a series of beta regressions with random effects using Proc Glimmix in SAS [7]. All effects were replicated except for the assessment type × slope × employment three-way interaction, which was not statistically significant in the beta regression (*P*<.35). These results suggest that our findings are generally robust across these two modeling choices. Beta regressions are frequently difficult to estimate especially with random effects [8,9] and replication of these results with beta regressions often did not converge, requiring a simplified random effect structure. This along with the cumbersome interpretation of logit transformed coefficients led us to proceed with the linear mixed model results in our main analysis.

**References**

1. Hawk LW Jr, Ashare RL, Lohnes SF, et al. The effects of extended pre-quit varenicline treatment on smoking behavior and short-term abstinence: a randomized clinical trial. Clin Pharmacol Ther 2012; 91(2): 172-180.

2. Hughes JR. Minnesota Tobacco Withdrawal Scale. 2017. http://www.med.uvm.edu/behaviorandhealth/research/minnesota-tobacco-withdrawal-scale [accessed 2023-04-28]

3. Muth ER, Stern RM, Thayer JF, Koch KL. Assessment of the multiple dimensions of nausea: The Nausea Profile (NP). J Psychosomatic Res 1996; 40(5): 511-520.

4. Younger J, Gandhi V, Hubbard E, Mackey S. Development of the Stanford Expectations of Treatment Scale (SETS): a tool for measuring patient outcome expectancy in clinical trials. Clin Trials 2012; 9(6): 767-76.

5. Gwaltney CJ, Metrik J, Kahler CW, Shiffman S. Self-efficacy and smoking cessation: a meta-analysis. Psychol Addict Behav, 2009. 23(1): 56-66.

6. Cappelleri JC, Bushmakin AG, Baker CL, Merikle E, Olufade AO, Gilbert, DG. Confirmatory factor analyses and reliability of the modified cigarette evaluation questionnaire. Addict Behav 2007; 32(5): 912-923.

7. Preacher KJ, Curran PJ, Bauer DJ. Computational tools for probing interactions in multiple linear regression, multilevel modeling, and latent curve analysis. J Educ Behav Stat 2016; 31(4): 437-448.

8. Smithson M and Verkuilen J. A better lemon squeezer? Maximum-likelihood regression with beta-distributed dependent variables. Psychol Methods 2006; 11(1): 54-71.

9. Zimprich D. Modeling change in skewed variables using mixed beta regression models. Res Hum Dev 2010; 7(1): 9-26.
